# The role of probiotics in modulating cariogenic bacteria and oral health outcomes: A systematic review and risk of bias assessment

**DOI:** 10.1007/s44445-025-00103-1

**Published:** 2025-12-02

**Authors:** Rahayu Zulkapli, Faezah Sabirin, Anis Najihah Salleh, Nurul Zurain Fatiha Zulaidi, Iman Nabilah Abd Rahim, Muhammad Nazirul Mubin Aziz

**Affiliations:** 1 Faculty of Dentistry UiTM Campus Sungai Buloh, Universiti Teknologi MARA (UiTM), Sungai Buloh Campus, Jalan Hospital, 47000 Selangor, Sungai Buloh Malaysia; 2 Cardiovascular Advancement and Research Excellence Institute (CARE Institute), Universiti Teknologi MARA, Selangor, Malaysia; 3 Collaborative Drug Discovery Research (CDDR) Group, Faculty of Pharmacy, Universiti Teknologi MARA, Selangor, Malaysia; 4 Faculty of Dentistry, AIMST University, 08100 Bedong, Kedah Malaysia

**Keywords:** Caries, Demineralization, Enamel loss, Mineral loss, Probiotics

## Abstract

This systematic review aims to evaluate the efficacy of probiotic interventions in the prevention of enamel demineralization, with a specific focus on randomized clinical trials (RCTs). A comprehensive literature search was conducted in the Cochrane Library, PubMed, Scopus, and Web of Science electronic databases, covering studies published until April 2025. The review follows the Preferred Reporting Items for Systematic Reviews and Meta-Analyses (PRISMA) 2020 guidelines. Article selection and data extraction were independently conducted, with methodological quality evaluated using the Jadad Score. A total of 34 RCTs were included, with 17 studies scoring 5 and 8 studies scoring 3 on the Jadad scale, indicating moderate to high methodological quality. The majority of the trials involved children aged 1 to 15 years, while eight studies focused on adult cohorts aged 18 to 65 years. Intervention durations varied from 6 days to 2 years. The most commonly used probiotic strains were *Lactobacillus, Bifidobacterium,* or a combination of both, delivered via dairy-based products, lozenges, mouth rinses, or probiotic tablets. Twenty-seven research studies demonstrated a significant reduction in the main cariogenic pathogen, *Streptococcus mutans* (*p* < 0.05 – *p* < 0.001), while findings on Lactobacillus reduction, plaque index, gingival status, salivary pH, and buffering capacity were inconsistent across studies. Long-term studies (≥ 6 months) showed more sustained effects, emphasizing the need for continuous probiotic intake to maintain oral health benefits. The findings suggest that probiotic interventions may represent a potential approach for preventing enamel demineralization, primarily through their ability to reduce cariogenic bacteria and increase salivary pH.

## Introduction

Dental caries is one of the most prevalent multifactorial diseases, affecting individuals across all age groups. Despite being preventable with proper oral hygiene and effective preventive strategies, caries remains a significant public health concern. The development of dental caries is influenced by four essential factors: time, substrate, host, and cariogenic bacteria, such as *Streptococcus mutans* (*S. mutans*), *Streptococcus sobrinus* (*S. sobrinus*), and *Lactobacillus* spp. (Pitts et al. 2017). These bacteria exist within a complex oral microbiome, a dynamic microbial community that plays a crucial role in maintaining oral health (Marsh & Zaura 2017). However, an imbalance in this microbiome, often referred to as dysbiosis, leads to an overgrowth of cariogenic bacteria, increasing acid production and enhancing caries risk (Takahashi & Nyvad 2016). Among these bacteria, *S. mutans* plays a pivotal role in caries pathogenesis due to its capability to produce oral biofilm and metabolize sucrose into extracellular polysaccharides (EPS). This process enhances bacterial adhesion and contributes to the stability of the biofilm. The metabolic activity of these bacteria, fueled by dietary carbohydrates, leads to the production of organic acids, which lowers the oral pH from neutral (7.0) to below the critical threshold of 5.5. This acidification initiates enamel demineralization and serves as a key factor in the caries process and could potentially lead to oral dysbiosis (Giacaman 2018).

Current caries management strategies emphasize a combination of non-invasive and restorative approaches. Initial treatment focuses on non-invasive methods, such as fluoride therapy, bioactive materials, and sealants, which may help control the progression of caries and promote enamel remineralization. When lesions become cavitated, restorative interventions like fillings are required; however, these procedures are irreversible and may compromise tooth structure (Schwendicke et al. 2016). As a preventive measure, fluoride application through toothpaste or professional topical treatments is widely adopted. However, excessive fluoride intake poses risks such as dental fluorosis (Buzalaf 2018).

There is increasing evidence supporting the role of probiotics in modulating the oral microbiome, caries prevention, preserving tooth structure (Saha et al., 2012; Seminario-Amez et al. 2021), and reducing mineral loss (Hao et al. 2015; Saha et al., 2012). Probiotic strains exert their protective effects by competing with cariogenic bacteria for adhesion sites on tooth surfaces, thereby inhibiting the growth of harmful microorganisms, including *S. mutans* (Chatterjee et al. 2018). A recent review by Poorni et al. (2019) highlighted the role of certain *Streptococcus* species, such as *Streptococcus salivarius* and *Streptococcus dentisani*, in inhibiting cariogenic bacteria and promoting oral biofilm balance, thereby contributing to caries prevention. However, a broader examination of various probiotic strains is warranted to fully understand their potential in maintaining oral health. The World Health Organization (WHO) defines probiotics as live microorganisms that, when administered in adequate amounts, confer health benefits to the host (Hill et al. 2014). Overall, probiotics are recognized for their ability to enhance immune function, reduce allergy susceptibility, improve lactose tolerance, and lower serum cholesterol levels (Plaza-Díaz et al. 2019; Khalesi et al. 2014). Within the oral health domain, commonly used probiotic strains include Lactobacillus, Streptococcus, and Bifidobacterium, which can be consumed in various forms such as drinks, lozenges, ice cream, and curds (Martín-Cabezas et al. 2016).

In the context of enamel demineralization prevention, studies have reported that Lactobacillus strains can effectively reduce oral acidity and decrease *S. mutans* levels, particularly in children with a high risk of caries (Rodriguez et al. 2016). Several systematic reviews have explored the potential of probiotics in caries prevention, suggesting their beneficial role in modulating the oral microbiome and reducing cariogenic bacterial activity. For instance, a systematic review by Laleman et al. (2014) indicated that probiotics might antagonize mutans streptococci, contributing to caries prevention. Similarly, a more recent review by Meng et al. (2023) demonstrated that probiotics, particularly *Lactobacillus rhamnosus*, could reduce the incidence and progression of caries in preschool children. However, these existing reviews exhibit variability in study quality, the probiotic strains assessed, and the clinical outcomes measured, underscoring the need for a more comprehensive analysis. Notably, the review by Laleman et al. (2014) focused primarily on the antagonistic effects of probiotics against mutans streptococci, while Meng et al. (2023) concentrated on the efficacy of *Lactobacillus rhamnosus* in preschool children. In contrast, our systematic review aims to evaluate the effects of various probiotic interventions in preventing enamel demineralization by analyzing findings from randomized clinical trials (RCTs) and assessing the risk of bias in existing literature up to April 2025. This approach seeks to address gaps related to strain-specific efficacy, study design consistency, and long-term effects, providing a more holistic understanding of probiotics’ role in dental health.

## Materials and methods

This systematic review is registered with the PROSPERO International Prospective Register of Systematic Reviews (Identifier CRD42025632910). This review follows the standard as suggested in the Preferred Reporting Items for Systematic Reviews and Meta-Analyses (PRISMA) statement (Page et al. 2021).

### Search strategy

An electronic literature search in the Cochrane Library, Scopus, PubMed and Web of Science databases was conducted until April 2025, with the keyword combination of "enamel demineralization" OR "enamel demineralization" OR "enamel loss" OR demineralization OR demineralization OR "mineral loss" AND probiotics OR probiotic. The search strategy did not include any restrictions on publication year. Guided by the main research question of “Does probiotic intervention have an effect on enamel demineralization in clinical trials?”, studies were selected based on the population, intervention, comparison, and outcome (PICO) framework; (P) studies looking at enamel demineralization (I) involving probiotic interventions, excluding combination treatments. (C) RCT studies comparing an intervention group to a control group and (O) the Primary outcome of interest is the effect of probiotic intervention on cariogenic bacteria load, as well as other relevant factors such as salivary pH, buffering capacity, plaque index, gingival health, and caries incidence.

### Study selection

Articles were included if the following criteria were fulfilled: (1) the study assessed the effects of known probiotic strains or genera, (2) the study focused on enamel demineralization, (3) the study conducted was a randomized clinical trial (RCT), (4) the study involved only probiotic interventions, excluding combination treatments, (5) the study is a full article original research study and, (6) the study was written in English. Articles were excluded from the review if: (1) they were review articles, case reports, or letters to the editor, (2) the study assessed the effects of non-probiotics or combination interventions, (3) the study focused on demineralization of structures other than enamel, or (4) the study was not a randomized clinical trial (RCT). All citations retrieved from the search were imported into EndNote software (Version X9). The citations were systematically organized, and duplicate entries were identified and removed.

### Data extraction

Data were extracted independently by four reviewers. Any disagreements on inclusion of specific articles between the reviewers were resolved by discussion. Data from the included literatures were extracted into a standardized form. The relevant information from the included studies was tabulated accordingly. The extracted data focused on key aspects of each study, including (1) the study objective, (2) a brief description of the sample population, (3) the probiotic strain used, (4) the duration of the intervention, (5) the measurement parameters, and (6) relevant results. The primary outcome of interest was the effect of probiotic intervention on salivary pH and cariogenic bacteria counts, assessed through colony-forming units (CFU) in RCTs.

### Data analysis: Risk of bias assessment (RoB)

The risk of bias in the included research studies was assessed using the JADAD scale which is a 5-point scale that is commonly used to evaluate the quality of RCTs. If the RCT scored ≥ 3 points, which indicates that the literature has superior quality (Jadad et al. 1996). The quality of the RCTs was assessed for any possible bias using the JADAD scale. The assessment includes the randomization application, double blinding and record of any withdrawal or dropouts. The JADAD scale was used to assess the quality of the RCTs. Only literature with a JADAD score of 3 or more were considered in this review.

## Result

### Study selection

**Fig. 1:**
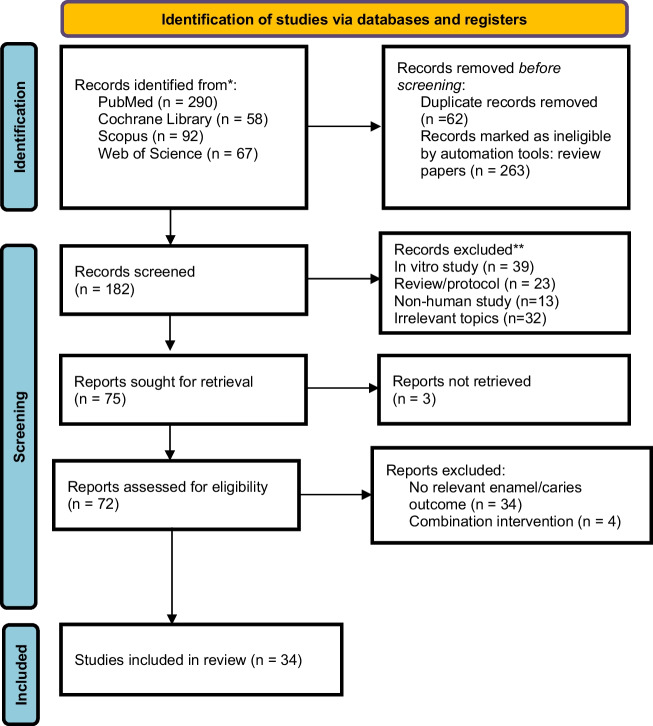
PRISMA Flow diagram of study selection process

The search strategy yielded 446 articles after removal of duplicates. Studies published up to April 2025 were identified through searches in the Cochrane Library (58), PubMed (290), Scopus (92), and Web of Science (67) databases. Based on pre-specified criteria, 72 full articles were retrieved, and 34 articles were then included in the qualitative synthesis (Fig. 1). Table 1 presents a summary of findings for all 34 included studies.

**Table 1:** Summary of selected RCT studies

Authors	Research Design	Probiotic Strain	Duration of intervention	Sample/Age	Measure	Results
Srivastava (2016)	Double Blind Parallel RCT	*Lactobacillus acidophilus* (Mother dairy b-activ Plus®, curd)	7 days	60 adults male and female 20- 25 years old	Salivary pH	↑mean salivary pH (*p* = 0.0067)
					*S. mutans* (CFU)	↓count (*p* = 0.0101)
Bhalla (2015)	RCT	*Bifidobacterium lactis* Bb12 (curd)	7 days	30 children 12- 14 years old	*S. mutans* (CFU)	↓count (*p* = 0.0001)
Zare Javid (2020)	Double blind RCT	*Bifidobacterium lactis* Bb12 (yogurt)	2 weeks	66 students 18–30 years old	*S. mutans* (CFU) *Lactobacillus*(CFU)	↓ *S. mutans* and lactobacillus counts (*p* = 0.001)
Chinnappa (2013)	RCT	*Bifidobacterium lactis* Bb12, *Lactobacillus acidophilus* La-5 (ice cream)	7 days	40 children 12–14 years old	*S. mutans* (CFU)	↓count (*p* = 0.001)
Sakhare (2021)	Prospective RCT	*Lactobacillus acidophilus* La-5, *Bifidobacterium lactis* Bb12 (mouth rinse)	24 days	57 children 6—12 years old	Salivary pH *S. mutans* (CFU)	No statistical difference in salivary pH ↓ *S. mutans* count (*p* < 0.05) After 24 days
Ashwin (2015)	Double-blind RCT	*Lactobacillus acidophilus La-5 Bifidobacterium lactis Bb-12* (ice-cream)	7 days (follow-up to 6 months)	60 children 6–12 years old	*S. mutans* (CFU)	↓ *S. mutans* (*p* < 0.001) after 7 days and 30 days; reduction not statistically significant at 6 months
Patil (2021)	RCT	*Lactobacillus acidophilus,* *Lactobacillus rhamnosus,* *Lactobacillus case,* *Lactobacillus bulgaricus,* *Lactobacillus plantarum,* *Bifidobacterium longum,* *Streptococcus thermophiles* (PrePro IBS, paste)	6 days	10 children 7–12 years old	*S. mutans* (CFU)	↓ *S. mutans* count (*p* < 0.05) between preintervention versus postintervention
Krupa (2022)	Double blind Parallel RCT	*Lactobacillus acidophilus-R,* *0052, Lactobacillus rhamnosus-R 0011, Bifidobacterium* *longum-R 00175, Bacillus coagulans-SNZ 1969,* *Saccharomyces boulardii* (Sporolac Plus, mouth rinse)	14 days	30 children 5 −12 years old 30 elderly > 60 years old	*S. mutans* (CFU)	↓count • Children (*p* = 0.023) • Elderly (0.018)
Sakaryalı Uyar (2024)	RCT	*Bifidobacterium breve,* *Bifidobacterium infantis, Bifidobacterium longum,* *Bifidobacterium bifidum* (NBL Probiotic Drop, drop)	6 months	58 children 2–12 years old	*S. mutans* (CFU) *Lactobacillus spp* counts (CFU)	↓ count (*p* = 0.01) The *Lactobacillus spp* counts was not statistically significant for both groups
Bolla (2023)	Double blind Parallel RCT	*Bacillus clausii* UBBC-07 (mouth rinse)	15 days	90 children 6–15 years old	*S. mutans* (CFU)	↓ *S. mutans* count (*p* < 0.001)
Lai (2021)	RCT	*Lactobacillus brevis* CD2 (lozenges)	60 days	68 diabetic children4-14 years old	Microbiological analysis Plaque pH Gingival status	↓ cariogenic bacteria in probiotic group (*p* < 0.01) Lowest pH changed (*p* < 0.01) and maximum pH fall (*p* < 0.01) ↓ bleeding score (p = 0.02) after 60 days
Campus (2014)	Double-blind RCT	*Lactobacillus brevis* CD2 (lozenge)	6 weeks	181 children 6–8 years old	Plaque pH (AUC5.7, AUC6.2) salivary MS (CFU) Bleeding on probing	↓ AUC5.7 & AUC6.2 (*p* < 0.01) ↓ MS counts (*p* = 0.01) ↓ bleeding (*p* = 0.02)
Ferrer (2020)	Double blind Parallel RCT	*Streptococcus dentisani* CECT 7746 (gel)	45 days	59 subjects 18–65 years old	*Plaque index (PI)* *Gingival index (GI)* *Salivary flow (SF)* *Microbial composition*	↓ PI ↓ GI ↑ SF (*p* = 0.05) ↑ *Streptococcus* (*p* = 0.021) Beneficial shift in oral microbiota composition at day 30 relative to baseline
Kang (2021)	Double blind RCT	*Weissella cibaria* CMU (tablet)	8 weeks	68 adults > 20 years old	*Cariview test (acidogenic potential of dental plaque)*	↓ Cariview score in both groups (*p* = 0.000); no between-group difference; probiotic group high-risk ↓ 32.4% → 8.8%, low-risk ↑ 0% → 52.9%
Staszczyk (2022)	RCT	*Lactobacillus salivarius* (tablet)	2 weeks	140 children 3–6 years old	Caries incidence Simplified Oral Hygiene Index (OHI-S)	↓ caries incidence (*p* < 0.001) No significant change in OHI-S
Janiani (2022)	Parallel RCT	*Lactobacillus casei* strain Shirota (LcS). (Yakult®, drink) Multiple species of *Lactobacillus* (enKor®-D, sprinkle on tongue)	7 days	34 children 3–6 years old	*S. mutans* (CFU) Plaque scores	↓ *S. mutans* count in Yakult® and enKor®-D (*p* < 0.05) ↓ Plaque score in Yakult®
Manmontri (2020)	RCT	*Lactobacillus paracasei SD1* (milk)	6 months	268 preschool children (3.2 ± 0.8 years)	*S. mutans* (CFU) Total lactobacilli	↓ *S. Mutans* count (*p* < 0.0033) ↑ lactobacilli (*p* < 0.0167)
Piwat (2020)	Double blind RCT	*Lactobacillus paracasei* SD1 (milk)	6 months	354 children 1–5 years old	Caries status using the modified Nyvad criteria	A decreased caries risk was shown in both the daily and triweekly probiotic groups
Wattanarat (2021)	RCT	*Lactobacillus paracasei* SD1 (milk)	6 months + 6 months follow-up	268 children 3–6 years old	*S. mutans* (CFU) *Lactobacillus* count salivary HNP1-3 caries progression	↓ *S. mutans* count (*p* < 0.001) ↑ Lactobacilli count (*p* < 0.001) ↑ HNP1-3 level (*p* < 0.001) ↓ Caries progression (daily & triweekly groups, *p* < 0.05)
Teanpaisan (2015)	Double-blind RCT	*Lactobacillus paracasei* SD1 (milk powder)	6 months (follow-up to 12 months)	122 children, 12–14 years old	*S. mutans* (CFU) *Lactobacillus* count Caries incrementt	↓ *S. mutans* at 3 months (*P* < 0.001) and 6 months (*P* = 0.001); ↑ lactobacilli (*P* < 0.001); ↓ caries increment in high-risk subgroup (OR = 4.55, *P* = 0.019)
Aminabadi (2011)	RCT	*Lactobacillus rhamnosus GG (yogurt)* ± *chlorhexidine*	2–3 weeks	105 children 6–9 years old	*S. mutans* (CFU) salivary LGG count	↓ *S. mutans* in CHX-only group (p < 0.001) and CHX + LGG group (steady up to 5 weeks) ↑ LGG in probiotic-only group (p < 0.001, not steady)
Cildir (2012)	RCT crossover	*Lactobacillus reuteri DSM 17938* + *L. reuteri ATCC PTA 5289 (drops)*	25 days × 2 intervention periods	19 children, 4–12 y (cleft lip/palate)	*S. mutans Lactobacillus* count	No significant reduction in *S. mutans* or LB (*p* > 0.05)
Alforaidi (2021)	RCT	*Lactobacillus reuteri DSM 17938* + *Lactobacillus reuteri ATCC PTA 5289 (drops in water)*	3 weeks	28 young adults with a mean age of 17.3 ± 1.1 years	Plaque pH *S. mutans* and lactobacilli counts	↑ plaque pH (*p* < 0.05) Statistically insignificant in *S. mutans* and lactobacilli counts (*p* > 0.05)
Borrell Garcia (2021)	Double blind RCT	*Lactobacillus reuteri* DSM 17938 (tablet)	28 days	27 adolescents 12–18 years old	*S. mutans* (CFU) *Lactobacillus sp* (CFU) Salivary pH	There were no statistically significant differences observed between the groups
Keller (2014)	Pilot RCT	*Lactobacillus reuteri* (tablet)	6 weeks	45 adolescents	ICDAS scoring	↓ ICDAS scores (no *p*-value stated; results described as not statistically significant)
Hasslof (2022)	Double blind Parallel RCT	*Limosilactobacillus reuteri* DSM 17938, *Limosilactobacillus reuteri* ATCC PTA 5289 (drop)	12 months	38 children 2–5 years old	*International Caries Detection and Assessment System (ICDAS)*	No statistical Difference between groups
Ghasemi (2017)	RCT	*Lactobacillus rhamnosus* (tablet)	2 weeks	44 children 6–12 years old	*S. mutans* level	↓ *S. mutans* in yogurt group (*p* = 0.001–0.01); gum group (*p* = 0.005–0.02)
Ratna Sudha (2020)	RCT	*Lactobacillus rhamnosus GG*	1 month	52 children3–5 years old	Caries status (ICDAS)	↓ *S. mutans* and LB in plaque and saliva (*p* < 0.05)
Nase (2001)	RCT	*Lactobacillus rhamnosus GG*	7 months	594 children 1–6 years old	Caries increment	↓ Caries in probiotic group (significant, *p* < 0.05; exact not stated)
Rodríguez (2016)	Cluster triple-blind RCT	*Lactobacillus rhamnosus SP1* (milk)	10 months	205 children 2–3 years old	Caries increment (ICDAS)	↓ Caries increment (ICDAS 2–6: *p* = 0.03; ICDAS 5–6: *p* = 0.02); logistic regression OR = 0.32 (*p* = 0.006)
Sandoval (2021)	RCT	*Lactobacillus rhamnosus SP1* (milk)	10 months	42 children 2–3 years old	ICDAS & hβD-3 levels	↓ hβD-3 in probiotic group (*p* = 0.0061); ↓ caries in control group (*p* = 0.0489); between-group *p* = 0.0140
Taipale. (2013)	RCT	*Bifidobacterium animalis subsp. lactis BB-12 (lozenge)*	21 ± 3.7 months	106 children, 4 years old	dmft index Caries prevalence	No significant difference in dmft or caries prevalence (*p* > 0.05)
Duraisamy (2021)	Double‑blind RCT	Multiple probiotics (Nilgiris Dahi Curd,curd)	15 days	40 children 6–12 years old	*S. mutans* (CFU)	↓ *S. mutans* count (*p* = 0.001) was observed in both groups treated with probiotics and synbiotics
Porksen (2023)	Double Blind Parallel RCT	*Lacticaseibacillus rhamnosus, LGG®, Lactobacillus paracasei subsp. paracasei, L. CASEI 431®* and *prebiotic arginine 2%* (lozenge)	10 −12 months	288 children 5–9 years old	Relative Risk Reduction (RRR) in dental caries transitions and lesion activity at the tooth surface level during	RRRs showed Less caries progression (*p* = 0.20), More regression (*p* = 0.44), Fewer active caries lesions (*p* = 0.15)

### JADAD score

Based on the JADAD score, 30 studies were classified as high-quality trials (≥ 3 points). All the trials clearly mentioned the appropriate protocols of randomization and blinding, with the fate of all patients also explicitly stated. While another 4 RCTs scored ≤ 3, since the details of random sampling and blinding were not clearly mentioned in the text (Table 2).

**Table 2:** JADAD score of selected RCT studies

List of Articles	Randomization			Blinding			An Account for all Patients		Total
	Mentioned in text	Appropriate method	Inappropriate method	Mentioned in text	Appropriate method	Inappropriate method	All patients included	Missing patient’s data recorded	
Srivastava (2016)	1	+ 1	0	1	+ 1	0	1	0	5
Bhalla (2015)	1	0	0	0	0	0	1	0	2
Zare Javid (2020)	1	+ 1	0	1	0	0	1	0	4
Chinnappa (2013)	1	0	0	1	0	0	1	0	3
Sakhare (2021)	1	+ 1	0	0	0	0	1	0	3
Ashwin (2015)	1	0	0	1	0	0	1	0	3
Patil (2021)	1	0	0	0	0	0	0	0	1
Krupa (2022)	1	+ 1	0	1	0	0	1	0	4
Sakaryalı Uyar (2024)	1	+ 1	0	1	0	0	0	0	3
Bolla (2023)	1	0	0	1	0	0	1	0	3
Lai (2021)	1	+ 1	0	0	0	0	1	0	3
Campus (2014)	1	0	0	1	0	0	1	0	3
Ferrer (2020)	1	+ 1	0	1	+ 1	0	1	0	5
Kang (2021)	1	+ 1	0	1	+ 1	0	1	0	5
Staszczyk (2022)	1	+ 1	0	1	+ 1	0	1	0	5
Janiani (2022)	1	+ 1	0	1	0	0	1	0	4
Manmontri (2020)	1	+ 1	0	1	+ 1	0	1	0	5
Piwat (2020)	1	+ 1	0	1	+ 1	0	1	0	5
Wattanarat (2021)	1	+ 1	0	1	0	0	1	0	4
Teanpaisan (2015)	1	+ 1	0	1	+ 1	0	1	0	5
Aminabadi (2011)	1	+ 1	0	1	+ 1	0	1	0	5
Cildir (2012)	1	+ 1	0	1	+ 1	0	1	0	5
Alforaidi (2021)	1	+ 1	0	0	0	0	1	0	3
Borrell Garcia (2021)	1	+ 1	0	1	+ 1	0	1	0	5
Keller (2014)	1	+ 1	0	1	+ 1	0	1	0	5
Hasslof (2022)	1	+ 1	0	1	0	0	1	0	4
Ghasemi (2017)	1	0	0	0	0	0	1	0	2
Ratna Sudha (2020)	1	+ 1	0	1	+ 1	0	1	0	5
Nase (2001)	1	+ 1	0	1	+ 1	0	1	0	5
Rodriguez (2016)	1	+ 1	0	1	+ 1	0	1	0	5
Sandoval (2021)	1	+ 1	0	1	+ 1	0	1	0	5
Taipale (2013)	1	0	0	0	0	0	1	0	2
Duraisamy (2021)	1	+ 1	0	1	+ 1	0	1	0	5
Porksen (2023)	1	+ 1	0	1	+ 1	0	1	0	5

### Primary outcomes

RCTs included in this study had a total of 1,834 salivary and plaque samples from volunteers. Of these, 1,486 samples were from children aged 2 to 15 years old, and 348 other samples were from adults aged 18 to 60 years old. Out of the 34 RCTs, 29 studies (85.3%) reported statistically significant reductions (*p* < 0.05) in *S. mutans* counts following probiotic intervention. There were, however, a few discrepancies among studies, as some reported substantial reductions, while others showed no statistically significant differences, particularly in relation to salivary pH, plaque scores, caries incidence, and gingival status. Various vehicles were used for probiotic delivery, including curds, ice cream, milk, tablets, and mouth rinses. These formulations incorporated one or more probiotic strains such as *Lactobacillus acidophilus*, *Lactobacillus paracasei*, *Bifidobacterium lactis*, and *Bacillus clausii*, among others (Table 1).

The primary outcome consistently assessed across the majority of the trials was the reduction in cariogenic bacterial load, especially *S. mutans* in saliva and dental plaque. Twenty-eight out of 34 RCTs (80%) reported a statistically significant reduction (*p* < 0.05 – *p* < 0.001), underscoring the potential of probiotics in modulating oral bacterial colonization associated with enamel demineralization.

### Secondary outcomes

Secondary outcomes included variations in salivary pH, buffering capacity, plaque index, gingival health, and caries incidence. Several studies observed an increase in salivary pH and buffering capacity post-intervention, suggesting a shift toward a less acidic environment, potentially lowering the risk of enamel demineralization. Nonetheless, results were inconsistent across the trials.

In terms of plaque accumulation and gingival inflammation, about 18 out of 34 studies (53%) reported improvements in gingival status or reduced plaque scores. Caries-related outcomes were assessed in 14 studies, with mixed findings; some demonstrated reduced caries development or slowed progression, while others reported no clear effect.

An additional secondary outcome explored was the long-term sustainability of probiotic effects. Studies with intervention durations of six months or longer were more likely to show persistent reductions in *S. mutans* and improvements in oral health indicators. This highlights the importance of consistent, long-term probiotic administration for maintaining oral benefits.

### Summary of reporting characteristics

Analysis of the included RCTs revealed methodological and reporting inconsistencies. While all 34 studies evaluated probiotic interventions for oral health, only 27 (79%) specified the probiotic strain(s) used, while all 34 studies (100%) clearly reported their intervention duration. Sample size details were incomplete in 7 studies, and only 16 (47%) described follow-up periods comprehensively. Reduction in *Streptococcus mutans* was the most frequently assessed outcome (32 research studies), yet statistical significance was reported in only 21. Secondary outcomes such as salivary pH, plaque index, gingival status, and caries incidence were addressed, with just 12 studies evaluating more than one secondary outcomes. These disparities in study design limit comparability across the selected RCT studies.

## Discussion

The studies included in this systematic review primarily assessed the efficacy of probiotic interventions in reducing cariogenic bacteria counts and their potential role in mitigating enamel demineralization. The findings consistently demonstrated that probiotics contribute to a significant reduction in cariogenic bacterial load, particularly *S. mutans*, which is a key driver of acidogenesis in the oral environment. By lowering *S. mutans* levels, probiotics help reduce acid production, thereby stabilizing salivary pH and enhancing buffering capacity. This shift toward a less acidic oral environment creates conditions that are less conducive to enamel demineralization, thereby promoting enamel integrity and overall oral health.

The findings from this systematic review provide robust evidence that probiotics contribute to the reduction of *Streptococcus mutans* counts, which plays a significant role in the caries process. While the included studies did not explicitly report this, the overall decrease in Streptococcus mutans counts observed across the research studies may be attributed to several biological mechanisms. Among others, one key pathway involves the production of bacteriocins, which are antimicrobial peptides, secreted by probiotic strains such as Lactobacillus and Streptococcus salivarius, which These peptides inhibit the growth of competing pathogens including S. mutans (Riccia et al. 2007). In addition, probiotics engage in competitive exclusion by occupying adhesion sites on tooth surfaces and within oral biofilms, thereby preventing the attachment and colonization of pathogenic species. Probiotics have also been shown to modulate the host immune response by enhancing salivary defense mechanisms. For example, certain strains stimulate the production of human neutrophil peptides (HNP1-3) and β-defensins, which are important antimicrobial components of innate immunity in the oral cavity (Sandoval et al. 2021; Wattanarat et al. 2021). Together, these mechanisms contribute to reductions in cariogenic bacterial load and improvements in oral health outcomes.

Enamel demineralization is, however, a multifactorial process influenced not only by bacterial colonization but also by the production of organic acids (Fejerskov et al., 2015), dietary carbohydrate intake (Moynihan and Kelly 2014), salivary composition (Lagerlof and Oliveby 2021), and host-related factors (Takahashi & Nyvad 2016). While probiotics may aid in modulating the oral microbiome and mitigating caries risk (Marsh and Zaura 2017), their efficacy should be considered within a broader preventive framework that includes dietary modifications, fluoride application, and overall oral hygiene practices (Pitts et al. 2017). Out of the 34 research studies reviewed, 21 of them reported on changes of on *S. mutans* colony-forming units (CFU) following probiotic interventions, among which 18 (86%) of them demonstrated notable significant reductions in the bacterial load, supporting the beneficial effect of probiotics in modulating the oral microbiome. Among the included studies that reported significant reduction in *S. mutans* count, as single- or multiple-strain interventions, all of them used *Lactobacillus acidophilus, Bifidobacterium lactis, Lactobacillus rhamnosus*, while probiotic interventions that used *Lactobacillus reuteri* (*n* = 3), as single or multiple strains, produced insignificant findings, which could be attributed to the short duration of the interventions (between 2 and 4 weeks) (Table 1). Other single strain probiotic interventions that significantly reduced the *S. mutans* count include *Bacillus clausii* UBBC-07, *Lactobacillus casei* and *Lactobacillus paracasei*. The findings from the majority of the included studies strongly suggest that probiotics may be effective in reducing cariogenic bacteria and modifying the oral environment. These findings were in agreement with Lin et al. (2017), demonstrating that various strains of lactobacilli inhibit the growth of *S. mutans* and change the composition of the bacterial biofilms. Although the antibacterial compounds produced by the probiotics were not reported in the included studies, probiotics exert their protective effect through competitive inhibition, bacteriocin production, and modulation of oral biofilm formation, which collectively suppress the growth of cariogenic bacteria (Xiao et al. 2018; Riccia et al. 2007).

In addition to their effect on *S. mutans*, some studies also investigated the reduction of *Lactobacillus spp* counts; and the majority (*n* = 4/7 studies) of those studies were presented with increases in *Lactobacillus spp.* count. Among the reviewed studies, Sakaryalı Uyar et al. (2024) found a significant reduction in *S. mutans* counts (*p* = 0.01) but no significant reduction in *Lactobacillus* spp. following probiotic intervention containing multiple *Bifidobacterium* species (without *Bifidobacterium lactis* Bb12) whilst, Zare Javid et al. (2020) observed a significant reduction in both *S. mutans* and *Lactobacillus* spp. counts (*p* = 0.001) after probiotic yogurt intake following intake of yogurt containing *Bifidobacterium lactis* Bb12. These findings suggest that while probiotics effectively reduce *S. mutans*, their effect on Lactobacillus remains uncertain, likely depending on the specific strains used, individual microbiome composition, and partly attributed to the different study duration between the two studies.

Beyond bacterial reduction, some included studies explored other oral health parameters, including plaque index (PI), gingival index (GI), salivary buffering capacity, and caries risk. Ferrer et al. (2020) and Janiani et al. (2022) reported a significant reduction in plaque and gingival index scores, suggesting that probiotics may contribute to better oral hygiene and reduced gingival inflammation. Similarly, Sakhare et al. (2021) found a significant reduction in *S. mutans* counts (*p* < 0.05) but no significant difference in salivary pH, indicating that not all probiotic strains influence pH regulation. However, Alforaidi et al. (2021) and Kang et al. (2021) observed a significant increase in plaque pH (*p* < 0.05) and a reduction in caries risk after probiotic use, suggesting that certain strains may help buffer the acidic environment, reducing enamel demineralization.

The duration of probiotic administration significantly influenced the effectiveness of *S. mutans* reduction. Studies with short-term probiotic administration (≤ 7 days) reported temporary reductions in *S. mutans* levels, whereas long-term studies (6–12 months) demonstrated sustained effects. Nase et al. (2001) emphasized the need for continuous probiotic consumption, as probiotics do not permanently colonize the oral cavity. This suggests that for optimal caries prevention, probiotics must be consumed regularly, similar to fluoride-based interventions (Riccia et al. 2007).

Although the results are encouraging, it is important to consider several limitations. The sample sizes in many studies were small, potentially reducing statistical power and affecting the generalizability of findings. Additionally, the studies used a variety of different probiotic strains, dosages, and delivery methods, making direct comparisons challenging. Another major limitation is that most of the studies were conducted in India among the Indian population, limiting the ability to generalize results across diverse populations. Future research should include multi-center randomized controlled trials (RCTs) with standardized probiotic formulations and diverse study populations to improve the reliability and applicability of findings.

Additionally, 11 studies reported a statistically significant increase in salivary pH, suggesting that some probiotic strains may contribute to a less acidic oral environment, reducing the risk of demineralization. Studies by Srivastava et al. (2016) and Alforaidi et al. (2021), demonstrated pH elevations (*p* ≤ 0.05), supporting the hypothesis that probiotic metabolites may help buffer oral acidity. However, the fact that only a minority of studies (11 out of 18) showed significant pH changes indicates that not all probiotic strains influence salivary pH to a clinically meaningful extent. This suggests that while probiotics effectively reduce *S. mutans* counts, their ability to modulate pH levels may depend on strain specificity and individual host factors (Huang et al. 2016; Jung et al. 2019).

The effectiveness of probiotics in reducing *S. mutans* counts was particularly notable in children, where the reductions were consistently greater compared to adults. Krupa et al. (2022) observed that probiotic interventions in children led to a significant decrease in *S. mutans* CFU (*p* = 0.023), whereas the reduction in adults was comparatively lower (*p* = 0.018). This finding aligns with existing literature indicating that probiotic adhesion is more effective in younger individuals, likely due to differences in oral biofilm maturity and salivary flow rates (Marsh et al., 2015; Slomka et al. 2017). These results suggest that age-related variations in the oral microbiome may influence probiotic efficacy, with younger individuals potentially benefiting more from probiotic interventions than older adults.

Among the 34 research studies reviewed in this systematic analysis, 30 studies received a JADAD Score of 3 or higher, indicating moderate to high methodological quality. These studies demonstrated robust study design, ensuring higher reliability of findings regarding the effects of probiotics on reducing *S. mutans* counts, modifying oral pH, and improving other oral health parameters. Studies with higher JADAD scores (≥ 4), such as Wattanarat et al. (2021) and Kang et al. (2021), provided stronger evidence supporting probiotics’ role in reducing *S. mutans* counts and increasing salivary pH.Additionally, the variability in intervention durations and inclusion of studies with lower methodological quality may introduce bias and confounding. These limitations highlight the need for more rigorously designed trials with consistent reporting and stratified analysis to confirm the durability and generalizability of probiotic effects.

The heterogeneity and reporting gaps identified in the included RCTs present a challenge to synthesize conclusive evidence. Incomplete reporting of intervention duration, sample characteristics, or statistical outcomes reduces the reliability and reproducibility of the findings. For example, without consistent descriptions of probiotic strains or follow-up periods, the dose–response relationship and long-term effects remain unclear. Additionally,, intervention duration varied widely across the studies, from 6 days to 24 months; yet the relationship between duration and outcome was not stratified or analyzed in detail. Short-term studies typically showed temporary reductions in S. mutans, whereas long-term studies demonstrated sustained effects. This introduces the possibility of temporal confounding, whereby the length of intervention may influence results but is not systematically accounted for. Future research should stratify outcomes based on duration to clarify the time-dependent efficacy of probiotics in oral health.

These deficiencies underscore the need for standardized reporting guidelines in probiotic trials related to oral health, such as adherence to CONSORT or PRISMA extensions for interventions. This review also notably highlighted that a considerable number of the included studies focused more on less invasive and feasible surrogate approaches, such as salivary pH and bacterial counts, in determining the effectiveness of probiotic interventions. This underscores a critical area for improvement in future trials, which should emphasize the need for more clinically meaningful outcomes, such as plaque pH, demineralization assays, or caries assessments; thus, providing more comprehensive evidence of probiotics as an alternative intervention. Addressing these limitations in future research would enhance comparability across studies and strengthen the evidence base for probiotic efficacy in caries prevention and oral health maintenance.

## Conclusion

The findings from this review strongly support the efficacy of probiotics in reducing *S. mutans* which plays a crucial role in caries prevention and enamel demineralization reduction. However, their effect on salivary pH modulation and *Lactobacillus* reduction remains inconsistent, and their long-term clinical impact is still inconclusive. Moving forward, well-designed large-scale RCTs with standardized probiotic strains, extended follow-up periods, and diverse populations are needed to confirm probiotics as a mainstream preventive strategy in dentistry. The variability in intervention durations and inclusion of studies with lower methodological quality may introduce bias and confounding. These limitations highlight the need for more rigorously designed trials with consistent reporting and stratified analysis to confirm the durability and generalizability of probiotic effects.

## Data Availability

All study related data are reported within the manuscript in the methods and results sections. Any further queries or clarifications may be sought by contacting the corresponding author.
